# Stitching a new garment: Considering the future of the speech–language therapy profession globally

**DOI:** 10.4102/sajcd.v69i1.932

**Published:** 2022-11-21

**Authors:** Bea Staley, Marise Fernandes, Ellen Hickey, Helen Barrett, Karen Wylie, Julie Marshall, Mershen Pillay, Harsha Kathard, Ryann Sowden, David Rochus, Carol E. Westby, T. Rosario Roman, Sally D. Hartley

**Affiliations:** 1 College of Health and Human Sciences, Charles Darwin University, Casuarina, Australia; 2 School of Health and Human Sciences, University of North Carolina, Greensboro, NC, United States of America; 3 School of Communication Sciences and Disorders, Dalhousie University, Nova Scotia, Canada; 4 Faculty of Health and Education, Manchester Metropolitan University, Manchester, United Kingdom; 5 Curtin School of Allied Health, Curtin University, Perth, Australia; 6 College of Health Sciences University of Ghana, Accra, Ghana; 7 School of Health Sciences, University of KwaZulu-Natal, Durban, South Africa; 8 Institute of Education, Massey University, Palmerston, New Zealand; 9 Discipline of Speech Language Pathology, University of KwaZulu-Natal, Durban, South Africa; 10 Department of Health and Rehabilitation Sciences, University of Cape Town, Cape Town, South Africa; 11 Bristol Medical School (Population Health Sciences), University of Bristol, Bristol, United Kingdom; 12 Yellow House Health and Outreach Services, Kisumu, Kenya; 13 Bilingual and Multicultural Services Albuquerque, Albuquerque, NM, United States of America; 14 School of Health Sciences, University of East Anglia, Norwich, United Kingdom

**Keywords:** communication disability, global practice, equity, future, Majority and Minority World contexts

## Abstract

Providing equitable support for people experiencing communication disability (CD) globally is a historical and contemporary challenge for the speech–language therapy profession. A group of speech–language therapists (SLTs) with ongoing and sustained experiences in Majority and Minority World contexts participated in five virtual meetings in 2021. The aim of these meetings was to develop provocative statements that might spur a global discussion among individuals and organisations that support people experiencing CD. The following questions were discussed: What is our vision for the future of the profession globally? What are the global challenges around access to speech–language therapy services?

Four main themes emerged: (1) the need to centre people experiencing CD as the focal point of services, (2) participation, (3) equity and (4) community. The themes relate to the need for a process of de-imperialism in the profession. Suggestions were made to develop more suitable terminology and to establish a global framework that promotes more equitable access to communication services. We seek the adoption of approaches that focus on reciprocal global engagement for capacity strengthening. Alternative models of culturally sustaining and equitable service delivery are needed to create impact for people experiencing CD, and their families worldwide.

**Contribution:**

Provocative statements were developed to prompt global conversations among speech–language therapy professionals and associations. We encourage readers to consider the questions posed, share their viewpoints and initiate positive change towards a global strategy.

## Introduction

We will not go back to normal. We are being given the opportunity to stitch a new garment. One that fits all of humanity and nature.

Sonya Renee Taylor

Global healthcare inequities predate the coronavirus disease 2019 (COVID-19) pandemic, but these inequities were illuminated and worsened by the pandemic, particularly for people who experience disabilities. The medicalisation of disability has been reinvigorated, resulting in calls to revisit the biopsychosocial model of disability. Health inequities are more obvious in Majority World countries, which are under-resourced countries where most of the world’s population live. This contrasts with the relatively small and privileged population living in high-resourced contexts in the Minority World (e.g. Canada). The Majority World contexts also exist within the Minority World countries, for example, for Aboriginal and Torres Strait Islander people in Australia. Most people experiencing communication disability (CD) live in the Majority World, and issues around equitable communication support are both a historical and a contemporary challenge (Pillay & Kathard, 2018).

An estimated 7% – 10% of the world’s population experience CD, which is extrapolated from estimates that 30% – 50% of the population who seek community rehabilitation or special education services experience CD (Hartley & Wirz, 2002). The term ‘people experiencing CD’ acknowledges that those with communication impairment(s) experience varying degrees of disability because of the dynamic interplay between their communication skills; health condition; social, physical and legal environments; and personal and contextual situations (such as support networks or individual resilience). In Minority World countries, speech–language therapists (SLTs) are the key service providers for people experiencing CD. Ratios of trained SLTs to population are relatively high in Minority World compared to Majority World countries, where formal speech–language therapy training is either nonexistent or has only recently been established (Pillay & Kathard, 2018; Wylie, McAllister, Davidson, & Marshall, 2018). Stringent standards of training at the university level, certification and/or licensure of SLTs by regulatory bodies result in highly qualified but few specialised professionals. At current rates of training SLTs, it would take several 100 years to address the population need in the Majority World.

Simply creating more training programmes in the Majority World is insufficient. The curricula and knowledge for training SLTs in the Majority World are largely exported from the Minority World, with few modifications (Wickenden, Hartley, Kariyakaranawa, & Kodikara, 2003). Such attempts to duplicate a profession in different cultural, economic and linguistic contexts need challenging (Hyter, 2014). The prioritisation of knowledge and practices from Minority World settings may result in services that are neither relevant nor culturally responsive. A possible solution in Majority World contexts is to build a workforce with a wider range of skills, including the capacity to support people experiencing CD (Pillay & Kathard, 2018; Wylie et al., 2018).

Concerns around inequities and the limited development of services to meet the global needs of people experiencing CD prompted the authors to hold a global virtual retreat to discuss the following questions: what is our vision for the future of the speech–language therapy profession globally? What are the challenges around global access to speech–language therapy services? The aim in this article was to develop provocative statements that might spur a global discussion among individuals and organisations that support people experiencing CD. The authors seek to inspire innovation to reduce inequities and ensure communication as a human right (McLeod, 2018).

## Creating a space for discussion

In late 2020, Bea Staley and Sally Hartley invited 15 colleagues to participate in this virtual forum. Thirteen people participated in the virtual retreat and in the subsequent writing of this article. Among us, this authorship group has decades of experience working and collaborating across Minority and Majority World contexts. Four of the participants or authors are from Majority World countries (South Africa, Uganda and Bolivia). The other nine have significant experience living and working in at least one Majority World country in sub-Saharan Africa, South America, Asia and/or the South Pacific. Five two-hour discussions were held on Zoom in January 2021. The schedule was created so that participants could attend three to five of the meetings, depending on their time zone. During the meetings, participants shared their perspectives and brainstormed ideas that could underpin a more equitable global strategy. Meetings were recorded and contemporaneous notes were taken. Although participants varied across meetings, the conversation was treated as a single narrative, with summaries at the beginning and end of each meeting. Recordings were available for those meetings that individual participants were unable to attend. Recordings and notes were used to develop themes and suggest principles.

## Themes

Participants recognised that consensus might not be reached on each topic, nor should consensus be a goal. For example, there exists disagreement among authors about the neoliberal roles played by the United Nations and affiliated agencies, such as the World Health Organization and the World Bank Group. Nonetheless, four recurrent major themes were identified from the narratives: (1) the need to centre people experiencing CD as the main focal point of services, (2) participation, (3) equity and (4) community. These themes were discussed and further refined in a series of collective writing groups to develop provocative statements for change. A summary of each theme is provided.

### Centring people experiencing communication disability

As a group, the authors noted that the conversation often converged on their own values, needs and beliefs as professionals and clinicians, instead of on those of the individuals and families that they work with. There is a need to centre people experiencing CD, recognising communication as both a human right and an essential function of being human (McLeod, 2018). Furthermore, the disorder-specific language the present authors use to identify people experiencing CD tends to utilise a deficit model, despite intentions to centre the person. For example, people with ‘speech sound disorders’, or ‘dysphasic’ and ‘stammerer’, are all examples of highly specific descriptors that largely centre on health conditions or on body structures and functions. The authors concluded that the term ‘people experiencing CD’ helps us better recognise and imagine the overall need for services to support people experiencing communication disabilities.

The experience of disablement by an individual is a complex synthesis of elements, such as the communication environment, accommodations made by communication partners and/or the perceptions of the individual. These are represented in the *International Classification of Functioning, Disability, and Health* (ICF; WHO, 2001) as a dynamic interaction among the key components of body functions and structure, activities and participation, and personal and environmental factors (physical and social). These experiences are unique to each person; therefore, each of these components and their interaction need to be addressed to maximise the efficacy of communication-related services and support. Arguably, the experience of CD and the resulting needs and wishes must be recognised as the main concern for speech–language therapy as a profession.

### Participation

While the ICF has been adopted nominally by national speech–language therapy organisations and many training programmes in the Minority World, the profession continues to focus on specific communication disorders. Speech–language therapy in the Minority World prioritises the medical model of rehabilitation, with assessments typically focused on identifying specific deficits in speech, language or swallowing functions. Intervention tends to centre around tasks intended to reduce the perceived deficit by increasing specific speech and language skills (e.g. increasing utterance length or articulatory precision), rather than attending to the necessary functional skills needed for participation at home or in community contexts. In the past two decades, there has been some movement in the Minority World towards life participation approaches to services by reducing barriers or improving support for participation, but these approaches are still considered fringe. A notable exception is the area of augmentative-alternative communication that has always kept participation at the fore. Alternative approaches focus more on participation at a societal level (Pillay & Kathard, 2018).

To improve life participation of people experiencing CD worldwide, services that uniquely support communication within specific communities need to be offered. For example, for people experiencing CD, participation could be enhanced through SLTs’ engagement with existing frameworks and models, such as community-based rehabilitation (CBR) or community-based inclusive development (CBID). Community-based rehabilitation aims to enhance the lives of people experiencing disability by ensuring inclusion and participation (WHO, 2010). Speech–language therapists in Majority World contexts where CBR services exist (e.g. Brazil, India and South Africa) have an important role to play in capacity strengthening in collaboration with CBR workers.

### Equity

Equitable speech–language therapy services demand systems that achieve *fairness* in access to effective communication-related support for people experiencing CD globally. While equality is giving everyone the same opportunity or the same treatment, equity is giving people what they need to succeed by providing appropriate adjustments and support. Equity necessitates consideration of race, gender and other social determinants of health in our frameworks for practice (Pillay & Kathard, 2018). Equitable services need to target varied dimensions of disability, from individual treatment focusing on communication disorders to communication partner training that supports life participation to systemic changes that support inclusion. Models that support broader community engagement must be developed for equitable service provision to become a reality. Examples include advocacy and prevention activities to reduce systemic barriers (Pillay & Kathard, 2018). Given that communication, language and culture are intricately intertwined, importing Minority World approaches into Majority World contexts is unlikely to result in equitable speech–language therapy service provision. Equitable services must be personally relevant, culturally sustaining, flexible and holistic. The following examples demonstrate the innovation necessary to develop equitable services in regions where SLTs are scant. In Western Kenya, a service model originally developed in Rwanda was adapted to better meet the needs of children with disabilities. ‘Communication Camps’ were created to empower families to take the lead in facilitating their children’s communication skills and participation in daily life with close support from Parent Liaison Officers (Staley, Hickey, Rochus, Musasizi, & Gibson, 2021). In Eastern Kenya, community consultations were held to determine the needs of people experiencing CD. Some groups then developed advocacy programmes that utilised local singing and dancing groups, which were the traditional mechanism for community education and public health messages (Hartley, Murira, Mwangoma, Carter, & Newton, 2009). Finally, in Rwanda, working with the United Nations High Council on Refugees, the refugee population selected and adapted a programme from the United Kingdom (UK) to increase communication accessibility in the community. The programme was then delivered by and to members of the community (Marshall et al., 2021).

### Community

Speech–language therapists must work collaboratively with community stakeholders to build models of equitable service delivery that increase accessibility and inclusivity for people experiencing CD (Hartley & Wirz, 2002; Pillay & Kathard, 2018; Wylie et al., 2018). As illustrated in the examples above, this requires adapting to the contextual factors of local communities. Intervention and support must respond to the myriad of cultural variations in beliefs and values that facilitate (or inhibit) the ability of people experiencing CD to participate at home and in the community (e.g. Hyter, 2014; Westby, 2013). Thus, services based on the concepts of communication and disability will differ from one place to another.

Sustainability of services must also be considered and is often achieved by collaborating with invested community stakeholders, including those in formal or informal roles. Related professionals such as teachers, occupational therapists and physiotherapists sometimes assume responsibility to address the communication needs of people experiencing CD. Community members such as pastors, community elders, herbalists, traditional or indigenous healers and family members often provide substantial informal support for people experiencing CD. Community-based rehabilitation and CBID models are implemented through the combined efforts of people experiencing disability and their families and communities, as well as relevant government and non-government health, education, vocational, social and other services. These efforts to support people experiencing CD are met with varying success. Speech–language therapists need to involve themselves in enhancing this support through capacity strengthening (Hartley & Wirz, 2002; Pillay & Kathard, 2018; Wylie et al., 2018) and active participation and sharing of expertise (Barrett & Marshall, 2012).

## Provocative statements for moving forward

The themes discussed above were used to develop provocative statements that SLTs might use to actively seek change to ensure more equitable service provision for people experiencing CD. This study proposes the following three actions for the speech–language therapy profession globally.

### Terminology needs to change to centre people experiencing communication disability and reframe speech–language therapy globally

Language matters: the words we choose position our worldviews. For example, speech–language pathology denotes the profession is situated in the medical model by the use of pathology or pathologists. We also question the appropriacy of ‘speech–language therapy’ to describe the work we do, given that speech and language are only two aspects of communication. The term ‘therapy’ positions us as professionals who ‘fix’ or seek to make ‘normal’ the varied speech and language skills of individuals. What if we viewed variation in communication as valuable and focused on use, participation and function? What name would better reflect a profession whose primary role is to support and advocate for people experiencing CD? Communication therapists or CD specialists are possibilities but are terms that have their own limitations.

Our professional terminology (e.g. ‘treatment’) needs to be reviewed to reflect underlying concepts related to human rights and social models of disability. The act of addressing our client group as ‘people experiencing CD’ can affect a simple shift in focus and a significant reframing of the work we do. We advocate for centring people experiencing CD.

### A de-imperialised framework for global speech–language therapy needs to be established

There is a pressing need for a process of de-imperialism to establish a framework of practice generated through reciprocal global engagement and a genuine sharing of perspectives among stakeholders. If equitable access to communication-related support and services is our professional goal, and community is our context, then priorities around training and advocacy need re-assessment. This necessitates Minority World speech–language therapy organisations and training programmes relinquishing their power as consultants and experts and allowing Majority World practitioners to lead the change in their own countries. Majority World organisations, programmes and practitioners would then lead the application of communication-related knowledge to develop professional training and services that meet the needs of their context. The role of Minority World practitioners would be one of support.

If we genuinely want a global change in the profession, Minority World speech–language therapy organisations, practitioners and university programmes need to engage with diverse voices, frameworks and models, including those from other disciplines (e.g. disability studies and public health). The aim is to shift speech–language therapy practice everywhere away from medical models that focus on individuals and impairments towards greater engagement with social models that prioritise communities and contextual factors of disability.

### Co-designing services with communities

We advocate for developing communication services that focus on capacity strengthening by partnering with communities and adapting contexts to better support communication. Speech–language therapists need to listen to people experiencing CD about where and how they seek support, particularly in communities where speech–language therapy services are unavailable, minimal or inaccessible. We must also seek to understand why services are not sought where they are available; for example, within Minority World countries where Majority World contexts exist, here historical injustices create distrust in professionals or organisations.

In those communities and sectors without SLTs, cross-sectoral collaboration is essential to improve communication support and services (Hopf, Crowe, Verdon, Blake, & McLeod, 2021; Wylie et al., 2018). How we as a profession choose to engage with community stakeholders will influence how CD is understood and the type of advice and support available or needed in the community. Better community engagement would also facilitate more culturally responsive and culturally sustaining services. Embracing a full cadre of workers (community health care assistants, family members, parent liaison, etc.) would enhance availability of communication support and services. When utilising a global framework, specifics will likely differ in each country or context. Thus, partnering with community stakeholders to co-design services across multiple sectors is essential to achieve these goals (Hartley et al., 2009; Pillay & Kathard, 2018; Wylie et al., 2018).

## Conclusion

We are not the first to suggest the speech–language therapy profession needs a new way forward (see also Hopf et al., 2021; Khoza-Shangase & Mophosho, 2021). The COVID-19 pandemic has forced us to confront ongoing global inequities in health care, education and employment sectors. The time of lockdown allowed us to reflect on the world we currently live in versus the world we want. Now is a time for professional transformation, for professional leadership as communication specialists and for listening carefully to diverse global voices. We seek to engage reciprocally with colleagues and with other disciplines to ensure that all people who experience CD have access to culturally sustaining communication support and services and to ensure the necessary realisation of communication as a human right (McLeod, 2018). We all agree that change is necessary, but the methods of change are not yet agreed. Some of us look to larger institutions for leadership and guidance, while others believe that grassroots organisations are best placed to innovate. From our collective experience, change comes in a myriad of forms, using both top-down and bottom-up approaches.

Innovation is needed to develop equitable and sustainable services globally. An inclusive umbrella term that centres attention on all who may need access to communication support and services is required. Contextually responsive forms of communication support and service delivery and evidence for the benefits of these programmes are essential. We must embrace various ways of knowing and being, extending far beyond the Minority World’s domination and gatekeeping of knowledge.

This is a call for speech–language therapy practitioners, organisations and people who experience CD to become involved in re-imagining the profession. We encourage consideration of the following questions. What is your vision for the future of the speech–language therapy profession? What changes in the speech–language therapy profession are needed to support culturally sustaining and equitable service provision in your locale, and globally? We invite you to share your ideas so that our profession can move forward purposefully and collaboratively with communities and people experiencing CD everywhere to achieve equity of access to support and services that enable them to lead rich and fulfilling lives of their choice. Imagine the garment that we can stitch to fit all of humanity.

## References

[CIT0001] Barrett, H., & Marshall, J. (2012). Implementation of the world report on disability: Developing human resource capacity to meet the needs of people with communication disability in Uganda. *International Journal of Speech Language Pathology*, 15(1), 48–52. 10.3109/17549507.2012.743035 23190008

[CIT0002] Hartley, S.D., Murira, G., Mwangoma, M., Carter, J., & Newton, C.R.J.C. (2009). Using community/researcher partnerships to develop a culturally relevant intervention for children with communication disabilities in Kenya. *Disability and Rehabilitation*, 31(6), 490–499. 10.1080/09638280802061944 18720117 PMC3595511

[CIT0003] Hartley, S.D., & Wirz, S.L. (2002). Development of a ‘communication disability model’ and its implication on service delivery in low-income countries. *Social Science and Medicine*, 54(10), 1543–1557. 10.1016/S0277-9536(01)00136-8 12061487

[CIT0004] Hopf, S.C., Crowe, K., Verdon, S., Blake, H.L., & McLeod, S. (2021). Advancing workplace diversity through the culturally responsive teamwork framework. *American Journal of Speech Language Pathology*, 30(5), 1949–1961. 10.1044/2021_AJSLP-20-00380 34314257

[CIT0005] Hyter, Y.D. (2014). A conceptual framework for responsive global engagement in communication sciences and disorders. *Topics in Language Disorders*, 34(2), 103–120. 10.1097/TLD.0000000000000015

[CIT0006] Khoza-Shangase, K., & Mophosho, M. (2021). Language and culture in speech-language and hearing professions in South Africa: Re-imagining practice. *South African Journal of Communication Disorders*, 68(1), a793. 10.4102/sajcd.v68i1.793 PMC8252163 34082547

[CIT0007] Marshall, J., Barrett, H., Mukagatare, C., Muragijemariya, G., Anwar, S., Harris, C., … Hussein, N. (2021). *Meeting the needs of refugees who experience communication disabilities in Rwanda – Adapting the Communication Access UK (CAUK) training materials*. Paper presented at RCSLT conference, October, online.

[CIT0008] McLeod, S. (2018). Communication rights: Fundamental human rights for all. *International Journal of Speech-Language Pathology*, 20(1), 3–1. 10.1080/17549507.2018.1428687 29466094

[CIT0009] Pillay, M., & Kathard, H. (2018). Renewing our cultural borderlands: Equitable population innovations for communication (EPIC). *Topics in Language Disorders*, 38(2), 143–160. 10.1097/TLD.0000000000000151

[CIT0010] Staley, B., Hickey, E., Rochus, D., Musasizi, D., & Gibson, R. (2021). Successes and challenge of speech language therapy service provision in Western Kenya: Three case studies. *South African Journal of Communication Disorders*, 68(1), a838. 10.4102/sajcd.v68i1.838 PMC8517751 34636594

[CIT0011] Westby, C.E. (2013). Implementing recommendations of the world report on disability for indigenous populations. *International Journal of Speech Language Pathology*, 15(1), 96–100. 10.3109/17549507.2012.723749 23072497

[CIT0012] Wickenden, M., Hartley, S., Kariyakaranawa, S., & Kodikara, S. (2003) Teaching speech and language therapists in Sri Lanka: Issues in curriculum, culture and language. *Folia Phoniatrica et Logopaedica*, 55(6), 314–321. 10.1159/000073255 14573988

[CIT0013] World Health Organisation (2001). *International classification of functioning, disability and health*. Geneva: WHO.

[CIT0014] World Health Organisation (2010). *Community based rehabilitation: CBR guidelines*. 26290927

[CIT0015] Wylie, K., McAllister, L., Davidson, B., & Marshall, J. (2018). Communication rehabilitation in sub-Saharan Africa: The role of speech and language therapists. *African Journal of Disability*, 7, 338. 10.4102/ajod.v7i0.338 29707516 PMC5913782

